# Multivalent Effect of Defect Engineered Ag_2_S/g-C_3_N_4_ 3D Porous Floating Catalyst with Enhanced Contaminant Removal Efficiency

**DOI:** 10.3390/ijerph20021357

**Published:** 2023-01-11

**Authors:** Nan Zhou, Yanzhang Li, Jie Chen, Mingxin Song, Linlin Zhang

**Affiliations:** 1 School of Applied Science and Technology, Hainan University, Haikou 570228, China; 2 Key Laboratory of Agro-Forestry Environmental Processes and Ecological Regulation of Hainan Province, School of Ecology and Environment, Hainan University, Haikou 570228, China

**Keywords:** C_3_N_4_, Ag_2_S, photocatalysis, pollutant degradation

## Abstract

Chlorophenols, as a major environmental pollutant, enter water systems through industrial wastewater, agricultural runoff and chemical spills, and they are stable, persistent under natural conditions, and highly hazardous to water resources. The objective of this article is to prepare Ag_2_S-modified C_3_N_4_ three-dimensional network photocatalyst by calcination method to use photocatalysis as an efficient, safe, and environmentally friendly method to degrade chlorophenols. Ag_2_S/C_3_N_4_ has an excellent visible light absorption range, low band gap, effective separation of photogenerated charges, and active free radicals production, all of which make for the enhancement of photocatalytic degradation performance of the Ag_2_S/C_3_N_4_ system. Under the light irradiation (λ ≥ 420 nm), the photocatalytic degradation efficiency of 2,4,6-Trichlorophenol reach 95% within 150 min, and the stable photocatalytic degradation activity can still be maintained under different pH water environment and four degradation cycles. When Ag_2_S is loaded on ACNs, more photogenerated electrons are generated and subsequent reactions produce highly reactive groups such as •O_2_^−^ and •OH that will originally be able to continuously attack TCP molecules to degrade pollutants. Therefore, this study shows that the photocatalyst provides a novel research approach for realizing the application in the field of pollutant degradation.

## 1. Introduction

Environmental pollution is becoming increasingly serious and has become a global problem that plagues human development, especially water pollution [1,2,3]. Pollutants entering water systems through industrial wastewater, agricultural runoff and chemical spills have posed serious threats to plants and animals [4,5,6]. Among them, chlorophenols, as a major environmental pollutant, are stable under natural conditions and can exist in the environments for a long time, which has been classified as carcinogens by the United States Environmental Protection Agency (US-EPA) [7,8]. Chlorophenols is of particular concern to the environment due to its mutagenic and carcinogenic properties. Chlorinated phenols are widely distributed in the environment due to their use as intermediates in the synthesis of important pesticides and as pesticides themselves; the uncontrolled use and disposal of chlorophenols has had a serious impact on surface water quality. Chlorophenols is a chlorinated phenolic compound. Trichlorophenol has been detected in alarming concentrations in several rivers in different regions. It is also believed that Chlorophenols is produced as a by-product of industrial processes such as water disinfection. In some cases, uncontrolled plant effluent results in concentrations higher than the natural average [9,10,11]. Ramos-Ramírez [12] studies the structure of MgO-MgFe_2_O_4_ oxides with good photocatalytic degradation of Chlorophenols in aqueous solution and Ji [13] observed that the valence band holes (VB holes) of g-C_3_N_4_ play an important role in the degradation of chlorophenols under N_2_ gas environment and proposed a possible degradation pathway for Chlorophenols. Therefore, it is particularly significant to develop effective methods to remove these pollutants.

Photocatalysis is an efficient, safe, and environmentally friendly technology that has been used in CO_2_ reduction, H_2_O_2_ production, and organic pollutant degradation [14,15,16,17]. Several common photocatalysts, such as ZnO, TiO_2_, and C_3_N_4_, have appeared in environmental treatment [18,19,20,21]. C_3_N_4_ has been widely used due to its low preparation cost, controllable morphology, and excellent thermochemical stability [22,23,24,25]. However, the narrow light absorption range and the faster charge complexation problems seriously hinder its further wide application [26,27].

In recent years, different modification approaches have been developed to improve the charge separation problem of carbon nitride and enhance its photocatalytic activity, such as the preparation of different morphologies, the introduction of defect engineering, and the construction of heterogeneous structures [28,29,30,31]. Among them, compounding C_3_N_4_ with semiconductor materials to form heterojunction structures is an effective method for improving the photocatalytic activity of C_3_N_4_ [32,33,34,35,36,37,38,39,40]. Zhang et al. [41] compounded C_3_N_4_ with KOH, which slowed down the complexation rate of electron combination holes and greatly improved the generation of H_2_O_2_ under visible light irradiation. Li et al. [42] prepared P-C_3_N_4_/PS-C_3_N_4_ composites by a simple calcination approach to construct a well-matched band arrangement. This improves the efficient separation and transport of photogenerated electrons, and maintains the high oxidation of the holes and the excellent reduction performance of the electrons. Li et al. [43] prepared a 2D/2D C_3_N_4_/MoS_2_ heterojunction photocatalyst by the mechanical grinding method. The combination of MoS_2_ improved the concentration of photogenerated carriers and realized the effective separation of photogenerated carriers. And the hydrogen production efficiency can reach 385.04 μmol∙h^−1^∙g^−1^.

Ag_2_S, as a common silver-based material, has the advantages of simple preparation, excellent performance, and a narrow band gap, which is usually used as a cocatalyst to compound with other semiconductor materials [44,45]. Ag_2_S can be easily reduced to metal Ag under light, and Ag can be used as an electron trapping agent to contribute to the separation efficiency of photogenerated electrons, thus improving the photocatalytic activity of the system [46,47,48,49]. Di et al. [50] successfully prepared Ag_2_S/BiFeO_3_ Z-Scheme photocatalyst by co-precipitation method. Ag_2_S slowed down the compounding of electrons and holes in Ag_2_S/BiFeO_3_. Compared with Ag_2_S and BiFeO_3_, the degradation rate of methyl orange by Ag_2_S/BiFeO_3_ reached 97% after 4 h. Zhang et al. [51] deposited Ag_2_S nanoparticles on WO_3_ nanorods, which shortened the charge migration pathway without reducing the redox ability of the photogenerated electrons. Moreover, Ag_2_S/WO_3_ exhibited the H_2_ release efficiency of 32.9 μmol h^−1^, which was Roughly four times as high as Ag_2_S.

In this work, in order to use photocatalytic methods to degrade chlorophenols, which have long been difficult to degrade in natural water environments, three-dimensional Ag_2_S/C_3_N_4_ consisting of Ag_2_S and C_3_N_4_ in the uneven mass ratio were prepared by the calcination method. In addition, the crystal structure, chemical structure, and microstructure of Ag_2_S/C_3_N_4_ were studied. The construction of heterojunction promoted the quick separation of photogenerated electrons, and significantly improved the degradation performance of 2,4,6-trichlorophenol (TCP). It provides a novel strategy for C_3_N_4_ in the field of environmental pollution treatment.

## 2. Materials and Methods

### 2.1. Preparation of Ag_2_S

A certain quantity of thiourea was weighed and dissolved in deionized water. An appropriate amount of silver nitrate solution (S:Ag molar ratio is 1:2) was poured into a constant pressure droplet funnel and added to the above solution under magnetic stirring. The reaction solution was centrifuged, and washed with deionized water and anhydrous ethanol three times. The obtained solid was put in a vacuum oven at 60 °C and dried for 12 h. The dry, dark brown powder is Ag_2_S.

### 2.2. Preparation of C_3_N_4_

Referring to the preparation methods of C_3_N_4_ and complex made by Chen et al. [35] and Peng et al. [38], the preparation methods are designed as follows. A certain amount of cotton was completely immersed in a 15 g melamine solution, and the mixture was stirred magnetically for 1 h. Then the sample was freeze-dried to remove the solvent. The dried sample was heated in a nitrogen atmosphere of 550 °C for 2 h, and CN was obtained after cooling.

### 2.3. Preparation of Three-Dimensional Ag_2_S/C_3_N_4_ (ACNs)

**Figure 1:**
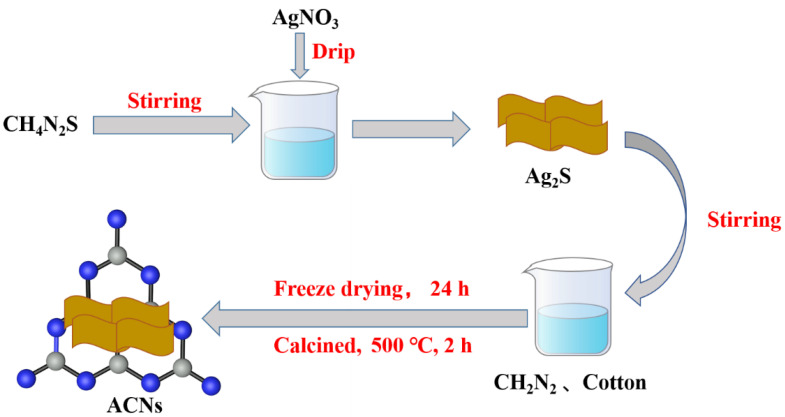
Schematic illustration of the preparation of ACNs.

As shown in Figure 1, there are detailed synthesis steps of three-dimensional Ag_2_S/C_3_N_4_ (ACNs). A certain amount of Ag_2_S powder was weighed and added to an appropriate amount of monocyanamide solution, and then ultrasonic was carried out to make Ag_2_S completely dispersed in the monocyanamide solution. An equal amount of cotton was added to the solution and continued to be ultrasounded until the solution was fully absorbed. Then the sample was freeze-dried for 24 h. The dried sample was put into a tubular furnace and calcined for 2 h at 550 °C under a nitrogen atmosphere to obtain three-dimensional Ag_2_S/C_3_N_4_ (ACNs). According to the different mass fractions of Ag_2_S, the different materials were named 10% wt% ACN, 30% wt% ACN, 50% wt% ACN, 70% wt% ACN, and 90% wt% ACN.

### 2.4. Characterization

X-ray diffraction (XRD) was obtained by a Bruker D8 Advance diffractometer (using Cu Kα radiation, λ = 1.54056 Å, 40 kV, 40 mA). The morphology and size of the resulting represented sample were investigated by using a field emission scanning electron microscopy (FE-SEM, Hitachi, Hitachi S-4800, Tokyo, Japan) and high-resolution transmission electron microscopy (HR-TEM, JEOL, JEM-2010, Tokyo, Japan). X-ray photoelectron spectroscopy was characterized by an X-ray photoelectron spectrometer (XPS, PHI-5700 ESCA, PHI, Chanhassen, MN, USA). The optical diffuse reflectance spectrum was conducted on a UV-vis-NIR scanning spectrophotometer (UV3600, Shimadzu, Kyoto, Japan) using an integrating sphere accessory.

### 2.5. Photocatalytic Degradation

Photocatalytic experiments were carried out by adding 50 mg of photocatalyst to 100 mL of a solution containing 2,4,6-trichlorophenol (TCP) (10 mg/L). Before the photocatalytic experiments, the solution containing the pollutants and the photocatalyst was placed in a dark room for 30 min to get the adsorption–desorption equilibrium. Then, the solution was irradiated under a 300 W xenon lamp. Every 20 min, 3 mL of each liquid sample was removed from the beaker and filtered with 0.22 μm Millipore filter heads for subsequent concentration tests [52,53].

## 3. Results and Discussion

### 3.1. Structure and Morphology of the ACNs

**Figure 2:**
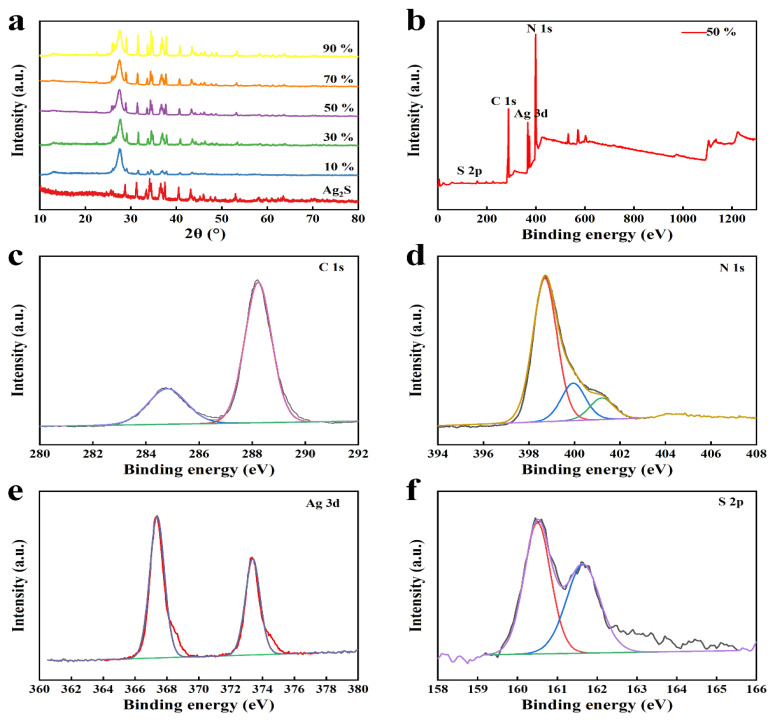
(**a**) The XRD patterns of Ag_2_S and ACNs. XPS spectra of 50% wt% ACN: (**b**) survey, (**c**) C 1s, (**d**) N 1s, (**e**) Ag 3d and (**f**) S 2p.

The crystal structure of ACNs was investigated by an X-ray diffractometer (XRD). Two types of carbon nitride were measured, and the results were presented As shown in Figure S1, there were two strong and sharp characteristic peaks at 13.01° and 27.22°, corresponding to the (100) and (002) crystal planes of C_3_N_4_ (JCPDS No. 87-1526). In Figure 2a, the diffraction peaks at 26.0°, 29.0°, 31.6°, 33.7°, 34.4°, 34.8°, 36.9°, 37.8°, and 40.8° were respectively assigned to (111), (111), (112), (120), (121), (022), (121), (103), and (031) crystal planes of monoclinic Ag_2_S (JCPDS 14-0072). For the ACNs, a diffraction peak was observed at 27.8°, which was the (002) crystal plane of g-C_3_N_4_. The characteristic peaks of Ag_2_S were observed at 31.6°, 33.7°, 34.4°, 34.8°, 36.6°, 36.9°, 37.2°, 37.8°, and 40.8°, and the diffraction peaks of Ag_2_S in ACNs gradually increased with the increase of Ag_2_S content. In addition, no additional impurity peaks were observed. The above results showed that ACNs with higher purity were successfully prepared.

To further analyze the chemical element composition and valence state of ACNs composites, 50% wt% ACN was taken as an example and analyzed by XPS. As shown in Figure 2b, 50% wt% ACN consisted of C, N, Ag, and S elements. In the C 1s spectrum (Figure 2c), the two characteristic peaks located at 284.8 eV and 288.1 eV corresponded to the C-C and sp^2^ hybrid carbon in n-C=N, respectively. In Figure 2d, the three characteristic peaks at 398.9 eV, 400.5 eV, and 401.7 eV corresponded to the C=N-C, N-(C)_3_, and C-N-H. In Figure 2e, two independent peaks situated at 367.8 eV and 373.8 eV were observed for Ag 3d_5/2_ and Ag 3_d/2_ in Ag_2_S. And the characteristic peaks at 162.2 eV and 163.4 eV in the S 2p spectrum ascribed to the S 2p_3/2_ and S 2p_1/2_ in Ag_2_S (Figure 2f). The above results indicated that ACNs composites have been successfully prepared.

**Figure 3:**
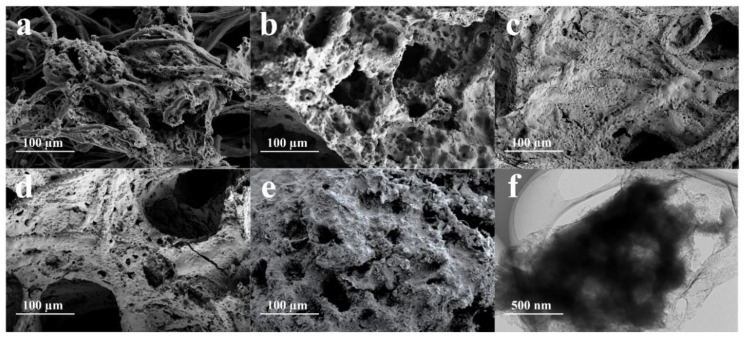
SEM images of (**a**) 10% wt% ACN, (**b**) 30% wt% ACN, (**c**) 50% wt% ACN, (**d**) 70% wt% ACN and (**e**) 90% wt% ACN, (**f**) TEM image of 50% wt% ACN.

The microstructures and morphology of ACNs were observed by SEM and TEM. As shown in Figure 3a,b, ACNs were a three-dimensional mesh material with a rough surface and porous structure. Ag_2_S was not completely wrapped on the surface, and C_3_N_4_ was still partially exposed. As for 50% wt% ACN and 70% wt% ACN, the content of Ag_2_S increases, but the three-dimensional structure of the ACNs material was still obvious, and no obvious aggregation of nanoparticles was observed on the surface (Figure 3c,d). When the mass fraction of Ag_2_S reached 90% wt%, the surface distribution of ACN was uneven and the agglomeration of large particles appeared obviously (Figure 3e). In addition, the TEM image of 50% wt% ACN showed that there were small sheets and holes on the surface (Figure 3f). The EDX mapping of 50% wt% ACN showed that it was composed of uniform distribution of C, N, Ag, and S elements.

### 3.2. Photocatalytic Removal of Pollutants

**Figure 4:**
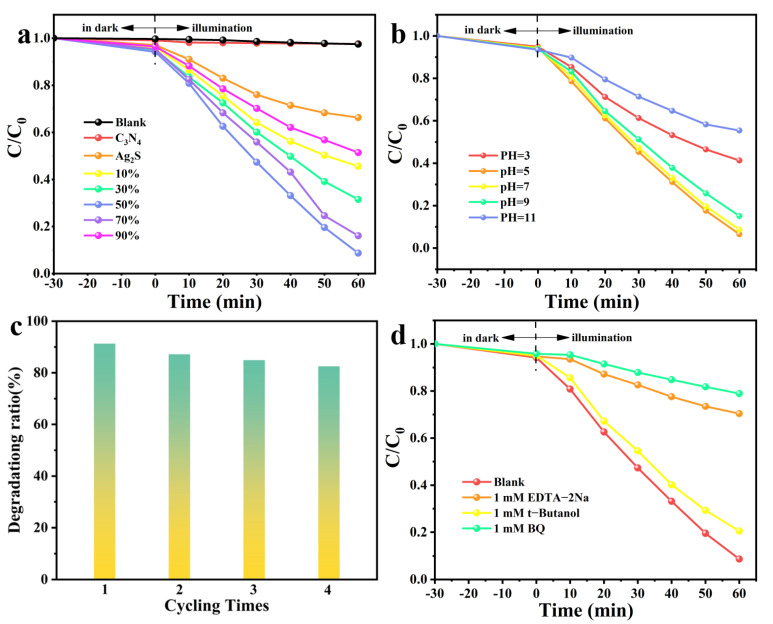
(**a**) Photocatalytic degradation of TCP under simulated sunlight irradiation. (**b**) Photocatalytic degradation of TCP in different waters under simulated sunlight irradiation. (**c**) Degradation rate and stability. (**d**) Photocatalytic degradation efficiency of TCP for 50% wt% ACNs with different quenchers.

The TCP degradation experiment was conducted to assess the photocatalytic performance of the ACNs (λ ≥ 420 nm), and the changes in UV-Vis absorption spectra of TCP solutions were used to monitor the photocatalytic degradation process of TCP. All samples were treated in dark for 30 min to reach adsorption equilibrium. As shown in Figure 4a, the blank sample hardly degraded TCP under simulated sunlight irradiation. the degradation efficiency of ACNs composites was apparently higher than that of Ag_2_S and C_3_N_4_, and the photocatalytic activity of ACNs gradually increased with the increase of the mass fraction of Ag_2_S in ACNs. Among them, the 50% wt% ACN could achieve the most excellent photocatalytic degradation efficiency of 91.2%. When the mass fraction of Ag_2_S exceeded 50% wt%, the photocatalytic degradation rate of the ACNs gradually declined. This indicated the composites formed by the appropriate amount of Ag_2_S and C_3_N_4_ could effectively contribute to the separation and transmission performance of photogenerated charges. However, the excessive amount of Ag_2_S caused the three-dimensional structure of the ACNs to be covered, and a large amount of particle agglomeration occurred, which reduced the photocatalytic capacity of the ACNs. In Figure 4b the TCP degradation process of 50% wt% ACN was investigated under different pH water environments. It was clear that different degrees of TCP degradation of 50% wt% ACN occurred at different pH conditions, and it showed the best degradation performance at pH 5. Since TCP is a weakly acidic compound, it does not dissociate at acidic pH values below the pKa of TCP, and dispersive interactions prevail. The higher binding mode of halogenated organic compounds prevents repulsive interactions between the activated carbon surface and TCP molecules, increasing the electrostatic attraction between TCP molecules and adsorption sites. However, at alkaline pH, TCP dissociates as a weakly acidic electrolyte, and electrostatic repulsion occurs between the negative charge in the solution and the chlorophenolate anion. There may also be competition between OH ions and TCP ions, which may reduce the removal rate of TCP. In general, protonated phenolics dominate at low pH and are more readily adsorbed than ionized phenolics. Besides, cyclic degradation experiments of TCP were performed to evaluate the stability of ACNs composites. As shown in Figure 4c, the 50% wt% ACN still maintained high degradation activity against TCP after four cycles (82.5%), indicating that the 50% wt% ACN had excellent stability performance.

To determine the crucial reactive oxygen species generated in the TCP degradation of 50% wt% ACN under visible light irradiation (Figure 4d). The EDTA-2Na, BQ, and t-Butanol were used to capture h^+^, •O_2_^−^, and •OH, respectively. When EDTA-2Na was added, 50% wt% © could degrade 29.6% TCP within 60 min. The degradation efficiency was 21.1% with BQ added, indicating that h^+^ and •O_2_^−^ played a key role in the TCP degradation of ACNs. Furthermore, with the addition of t-Butanol, 50% wt% © still degraded 79.5% TCP, showing that •OH was involved in the degradation process of TCP, but was not the main active substance.

### 3.3. Possible Photocatalytic Mechanism

**Figure 5:**
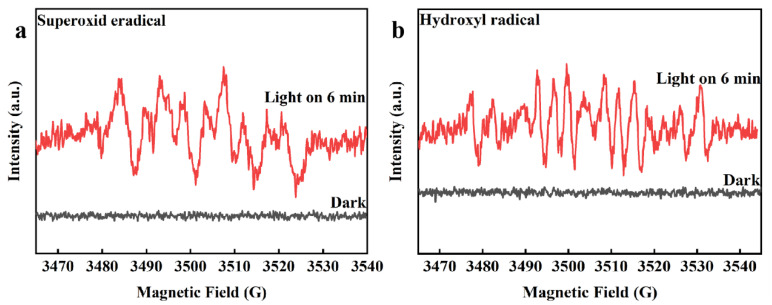
ESR spectra under dark and visible-light irradiation: (**a**) DMPO-•O_2_^−^ of 50% wt% ACNs, (**b**) DMPO-•OH of 50% wt% ACNs.

ESR was utilized to clarify the reactive oxygen species produced by ACNs to reveal the photocatalytic mechanism (Figure 5a,b). Taking 50% wt% ACN as an example, the signals of •O_2_^−^ and •OH were not tested under the dark, indicating that ACNs could not produce reactive oxygen species under this condition. Meanwhile, the characteristic peaks of DMPO-•OH and DMPO-•O_2_^−^ were detected under visible light. These results indicated that the ACNs could generate •O_2_^−^ and •OH under illumination. Combined with the live species capture experiment, the TCP degradation by ACNs was mainly dependent on •O_2_^−^, followed by h^+^, and the effect of •OH was minimal.

**Figure 6:**
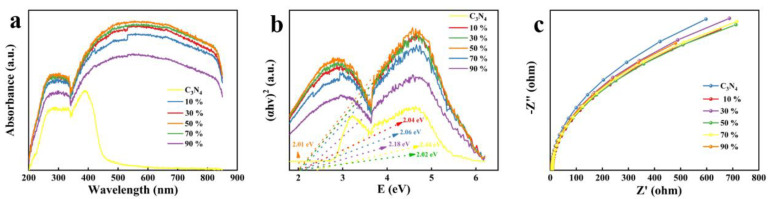
(**a**) UV-vis absorption spectra of ACNs. (**b**) Plots of the (ahγ)^2^ versus photon energy (hγ) of ACNs. (**c**) Electrochemical impedance spectroscopy of ACNs.

The separation and transport behavior of photogenerated carriers and the light absorption ability are inextricably linked to the photocatalytic performance of the ACNs, so ACNs were measured by UV-vis absorption spectra, band gap, and electrochemical impedance spectroscopy. As shown in Figure 6a, the ACNs exhibited excellent visible light absorption performance with the addition of Ag_2_S. According to the calculation, the band gap energies of 10% wt% ACN, 30% wt% ACN, 50% wt% ACN, 70% wt% ACN and 90% wt% ACN were 2.04 eV, 2.02 eV, 2.01 eV, 2.06 eV, and 2.18 eV, respectively (Figure 6b). Obviously, the band gap of 50% wt% ACN was the smallest. This may be because the introduction of Ag_2_S increased the light absorption capacity of the ACNs, thus reducing the band gap. In Figure 6c, the arc radius of 50% wt% ACN was smaller than that of other ACNs, showing the optimal charge separation performance. In conclusion, the 50% wt% ACN showed the best photocatalytic performance.

**Figure 7:**
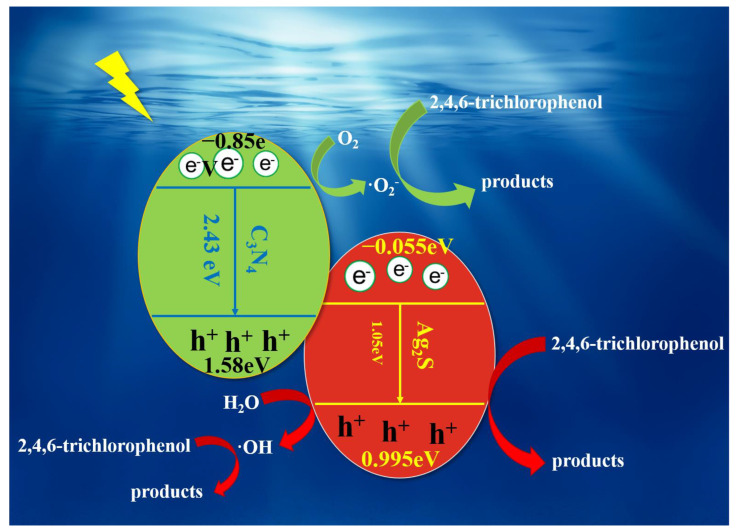
Schematic diagram of ACNs for degradation of pollutants.

Based on the above experimental results, a possible photocatalytic degradation mechanism was presented. As shown in Figure 7, In 50% wt% ACN, visible light transferred electrons (e^−^) from the valence band (VB) of carbon nitride to the conduction band (CB), while an equal number of electron holes (h^+^) were retained in VB. Due to the difference in band gap between silver sulfide and carbon nitride before, the electrons and holes generated by carbon nitride were transferred to silver sulfide after the compounding of the two materials, which greatly inhibited the compounding of electrons and holes in the materials and improved the This greatly inhibits the complexation of electrons and holes and improves the utilization of visible light, which in turn improves the photocatalytic performance of the material and the degradation of small molecule organics. The generated electrons and holes are transferred at the interface of the composite coating, and the photogenerated electrons react with oxygen to form •O_2_^−^, which is the most effective way to improve the photocatalytic performance of the material [54,55,56,57].

## 4. Conclusions

In summary, the three-dimensional Ag_2_S/C_3_N_4_ composite photocatalysts were successfully prepared by a simple calcination method. The addition of Ag_2_S broadened the visible light response capacity of the ACNs, reduced the band gap, and promoted the effective separation of electrons and holes. Among the ACNs, the 50% wt% ACN exhibited excellent photocatalytic activity, which could degrade 91.2% TCP within 60 min and maintain good photocatalytic activity under different pH of the water environment. In addition, the high photocatalytic activity of 50% wt% ACN remained stable after four degradation cycles. In this process, •O_2_^−^, h^+^, and •OH all contributed to the improvement of photocatalytic degradation performance. This work provides a novel solution strategy for pollutant degradation.

## Data Availability

The data presented in this study are available on request from the corresponding author. The data are not publicly available due to privacy issues.

## Supplementary Materials

The following supporting information can be downloaded at: https://www.mdpi.com/article/10.3390/ijerph20021357/s1, Figure S1: The XRD patterns of C_3_N_4_; Figure S2: Mapping diagram of 50 wt% ACN; Figure S3: Corresponding pseudo-first-order kinetic curves of photocatalytic degradation of TCP, reaction rate constants of photocatalytic degradation of TCP; Figure S4: Photocatalytic degradation of TCP by adding 50% wt% ACN with different trapping agents.
