# Supplementary material for: Multivalent Effect of Defect Engineered Ag2S/g-C3N4 3D Porous Floating Catalyst with Enhanced Contaminant Removal Efficiency
Source: Int J Environ Res Public Health. 2023 Jan 11;20(2):1357. doi: 10.3390/ijerph20021357 (PMC9859220; doi:10.3390/ijerph20021357)
Supplement: Supplementary file 1 [file ijerph-20-01357-s001.zip › ijerph-2080935-supplementary.pdf]

S1

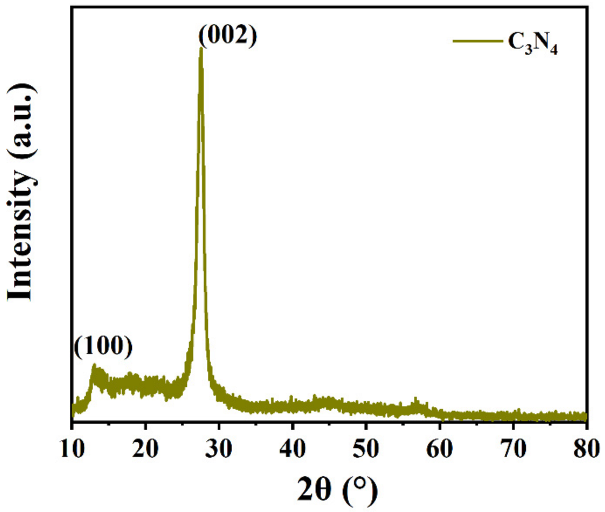

Figure S1. The XRD patterns of C_3N_4

S2

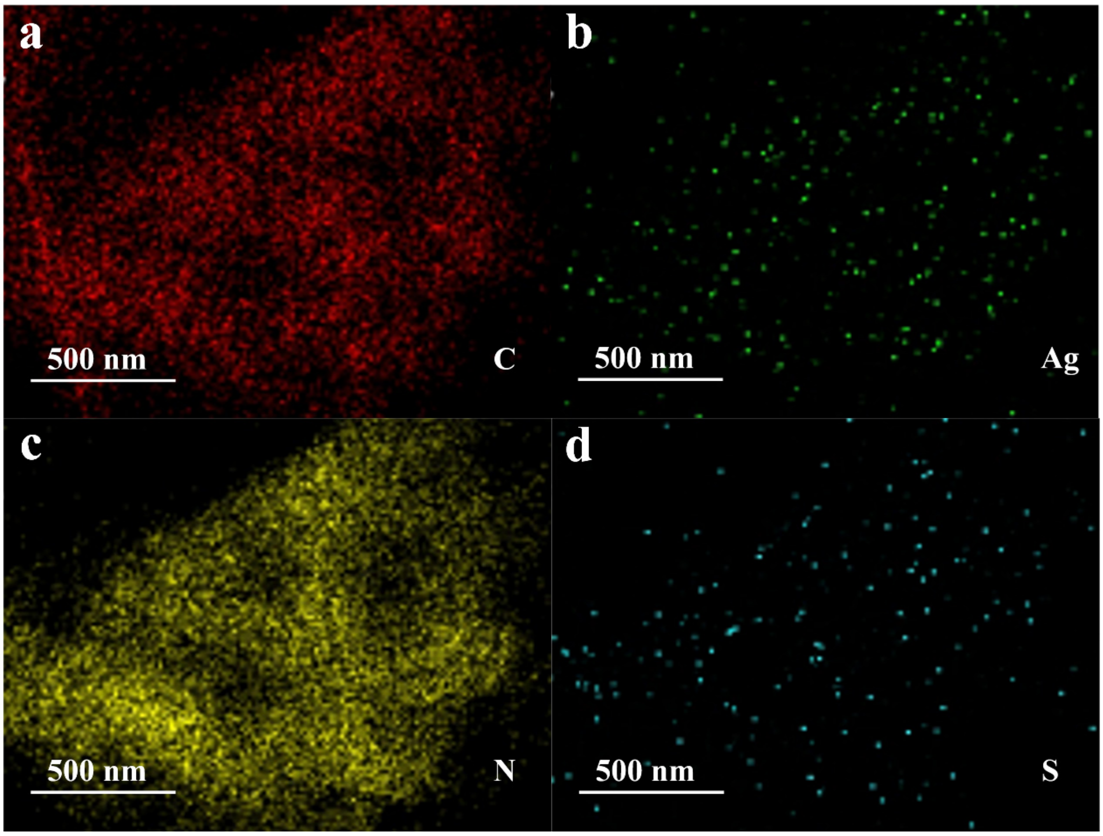

Figure S2. Mapping diagram of 50 wt % ACN.

S3

In Fig. S3a , all curves show a linear relationship indicating that the degradation behavior adheres to first-order reaction kinetics. According to the slope of the lines, the apparent rate constant (Kt) can be calculated. As shown in Fig. S3b and d, the Ag_2S/g- C_3N_4 ternary composite has the highest rate constant, which is 4.95 times higher than those of pure C_3N_4.

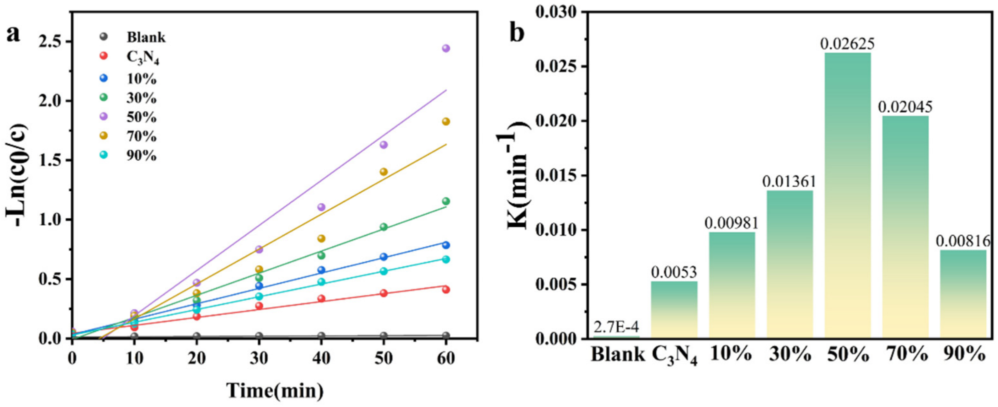

Figure S3. Corresponding pseudo-first-order kinetic curves of photocatalytic degradation of TCP, reaction rate constants of photocatalytic degradation of TCP

S4

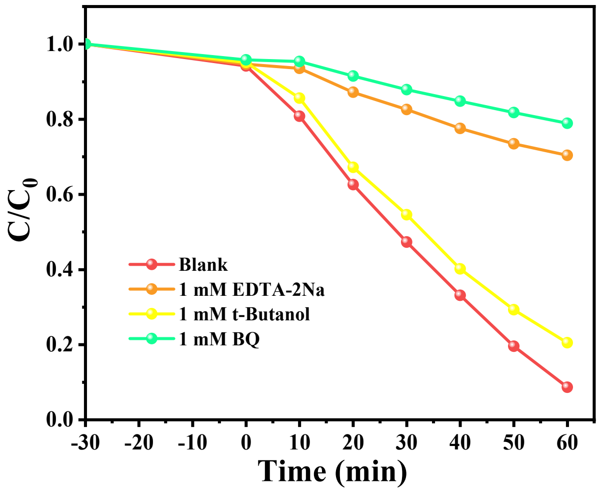

Figure S4. Photocatalytic degradation of TCP by adding 50% wt % ACN with different trapping agents

To determine the different roles of different active radicals in the degradation of TCP, the specific scavenger was used to conduct free radical capture experiments on corresponding active substances. The trapping agent BQ was used to scavenge $•O_2^-$, t-Butanol to scavenge •OH, and EDTA-2Na to scavenge electron h^+. In Fig. S4. the main

reactive oxygen species were h+ and $\cdot O_2^-$ in TCP degradation process, which were the main active substances that can improve the degradation ratio of TCP under visible light irradiation
